# Timing of diagnosis and factors associated with delayed detection of structural birth defects in children: a multicenter cross-sectional study from Addis Ababa, Ethiopia

**DOI:** 10.1136/wjps-2026-001186

**Published:** 2026-08-12

**Authors:** Hana Abebe Gebreselassie

**Affiliations:** 1 Surgery, St Paul’s Hospital Millennium Medical College, Addis Ababa, Ethiopia

**Keywords:** Child Health, Congenital Abnormalities, Neonatal Screening

## Abstract

**Background:**

Congenital birth defects contribute substantially to child mortality in the Global South. From the caregiver perspective, we aim to describe the timing of diagnosis of children with structural birth defects, where diagnosis is delayed, and the underlying reasons.

**Methods:**

A cross-sectional study of children with structural birth defects at three public hospitals in Addis Ababa was conducted using caregiver interviews and clinical chart review. Data were analyzed using SPSS V.26. Binary and multivariable logistic regression was used to identify factors related to the delayed diagnosis.

**Results:**

Totally, 423 children with structural birth defects were enrolled; 358 (84.6%) were male. Median age at the time of data collection was 2.12 years (interquartile range (IQR), 0.8–5.0 years). Among 441 diagosed defects, the three most frequent structural birth defects were hypospadias (133, 30.2%), undescended testes (95, 21.5%), and anorectal malformation (38, 8.6%). Among 77 children with prenatally detectable defects, 32 were diagnosed antenatally. A total of 411 (97.2%) of the mothers had antenatal follow-up. Among 362 facility-born children with externally visible or clinically detectable defects, 104 (28.7%) were diagnosed before discharge. Overall, the diagnosis of a structural birth defect was delayed in 274 (64.8%) children. The main reasons for this delay were lack of awareness, delayed referral, and false reassurance by a health professional. Being delivered at a health center (*p*<0.001, adjusted odds ratio (*aOR*)=2.17) was associated with a delayed diagnosis.

**Conclusion:**

There is a significant delay in diagnosing structural birth defects during both the antenatal and postnatal periods. These missed opportunities can be addressed through comprehensive screening programs, capacity-building sessions for healthcare workers, and raising public awareness.

**WHAT IS ALREADY KNOWN ON THIS TOPIC**

- Structural birth defects are a major contributor to under-five mortality in the Global South. In low- and middle-income countries, diagnosis is often delayed, but the extent of delay across various defects and the reasons for this delay from the perspective of caregivers have not been well quantified.

**WHAT THIS STUDY ADDS**

- This multicenter study in three teaching hospitals in Addis Ababa quantified both prenatal and postnatal delays in diagnosing structural birth defects and identified caregiver-reported reasons for late diagnosis. Of note, only one-quarter of newborns with clinically detectable malformations were diagnosed before discharge from health facilities.

**HOW THIS STUDY MIGHT AFFECT RESEARCH, PRACTICE OR POLICY**

- By highlighting where most antenatal follow-ups and births occur along with the magnitude of diagnostic delay and caregiver-identified barriers, this study underscores the need for newborn screening programs, capacity building for frontline healthcare providers, and public awareness initiatives. These findings provide actionable evidence to guide future research, to inform medical practice, and to inform health policy aimed at improving early detection of birth defects in Ethiopia and similar settings.

## Introduction

 Congenital birth defects are defined as structural or functional abnormalities that are present at the time of delivery.^1 2^ They are common health problems in low-income countries. Indeed, 60% of children born with congenital anomalies globally are from these countries.^3^ According to a report by the World Health Organization (WHO), 90% of children born with serious congenital disorders are in low- and middle-income countries (LMIC).^4^

While there are no population-based studies describing the actual prevalence of congenital birth defects in Ethiopia, healthcare workers experience these cases in everyday clinical practice. Moreover, a limited number of hospital-based studies have shown that congenital anomalies are among the most common causes of hospital admission.^5–7^

In addition to simple prevalence, congenital anomalies are important causes of under-five mortality and morbidity. For instance, 72% of deaths related to major congenital anomalies occur in low-income countries.^3^ According to a report by the Sub-Saharan African Network for Congenital Anomalies, in 2017, these were the fifth leading cause of death in children under 5 and were responsible for 584 900 deaths.^8^

In low-income countries, most congenital anomalies usually go undetected during pregnancy. In addition, major congenital anomalies are often missed after birth, even in neonates delivered in tertiary healthcare facilities due to the absence of neonatal screening programs. As a result of this delay in diagnosis, children in these regions suffer from unacceptably high rates of complications and preventable deaths.

Several factors contribute to the delayed diagnosis of congenital anomalies in the Global South. These include limited access to healthcare facilities, insufficient medical resources, the absence of screening programs, and a lack of trained healthcare professionals equipped to identify and manage these conditions promptly.

Congenital anomalies contribute to under-five mortality, although population-based Ethiopian data on structural birth defects remain limited; delays in diagnosis are rarely investigated. Existing studies highlight late detection of several birth defects; their extent and caregiver perspectives have similarly not been quantified. This multicenter study in three public hospitals in Addis Ababa, Ethiopia, examines both prenatal and postnatal diagnostic timing. It also explores caregiver-reported barriers, providing new evidence to guide early detection strategies in Ethiopia.

## Methodology

### Study design and participants

This study was conducted in three public hospitals in Addis Ababa, Ethiopia: St Paul’s Hospital Millennium Medical College, St Peter’s Specialized Hospital and Yekatit 12 Hospital Medical College. These facilities are teaching and referral hospitals and deliver healthcare services to patients from across Ethiopia. They also provide several specialty care services, including pediatric surgery.

This was a cross-sectional study that enrolled children diagnosed with a structural birth defect who were evaluated in the pediatrics service areas of the three listed public hospitals. Sample size was calculated using the single population proportion formula, considering a proportion of 50%, as there was no similar study with a comparable set-up. In addition, the critical value set was 1.96, which corresponds to the 95% confidence interval (*CI*); the margin of error was 5%. Sample size was also calculated using OpenEpi (V.3.01). Assuming a 95% confidence level, 80% power, a 1:1 ratio of exposed to unexposed, and an expected prevalence of delayed diagnosis of 50%, the minimum required sample size was 370. After adjusting for a 10% non-response, the final sample size was 407. The higher sample size of 423 was used in this study.

Sample size was divided among the three public hospitals proportionally based on the number of patients seen in the pediatrics clinics over the previous year at each institution. At each study site, children with structural birth defects were enrolled consecutively until the allocated quota of each institution was fulfilled. All children aged under 18 years who attended the study facilities during the study period were approached for inclusion in the study.

### Data collection and definition

Sociodemographic and clinical profile data of children with structural birth defects were collected through interviews using a structured checklist and also through chart review by trained data collectors. Bias was minimized through standardized data collection tools, training of interviewers, and validation of chart reviews. There were no missing data.

Operational definitions of delayed diagnosis. (1) For structural birth defects that are detectable by prenatal ultrasound, delayed diagnosis was defined as a diagnosis not made before birth. This definition was used because fetal interventions are not available in the study sites. Moreover, the purpose of antenatal detection is mainly for planning the termination of pregnancy or early postnatal intervention and parental counseling. (2) For structural birth defects that are overt, physically detectable, or with external stigmata for an underlying birth defect, delayed postnatal diagnosis was defined as a diagnosis not made before discharge of the newborn for children born at a health facility. (3) For children with structural birth defects and whose mothers did not have antenatal follow-up or those who were not delivered in health facilities, delayed diagnosis was defined as follows: for children with structural birth defects that cause early symptoms or are externally visible, diagnosis beyond 1 week of age; for children with structural birth defects with no external signs or not symptomatic, diagnosis beyond neonatal age (28 days of life).

The thresholds used to define delayed diagnosis were selected to reflect the realities of clinical practice in Ethiopia. They were not derived from formal international guidelines but rather from consensus among consultant pediatric surgeons at our institution, who considered them most appropriate for the local context. We acknowledge that incorporating the place of delivery into the operational definition could introduce differential misclassification. To minimize this risk, the defined thresholds were applied consistently across all cases. The same operational rules were used regardless of outcome status, ensuring uniformity in classification.

A detailed description of prenatally detectable, physically detectable structural birth defects, as well as those that present with early and late signs and symptoms, is provided in online supplemental file 1. In addition, a case-by-case definition of delayed diagnosis for the structural birth defects described in this study is illustrated in online supplemental file 2.

### Statistical analysis

Data were analyzed using SPSS V.26. Frequencies and percentages were used to describe categorical variables, while mean±standard deviation (SD) or median and interquartile range (IQR) were used to describe continuous variables.

Binary logistic regression was used to assess the association of different factors with the delayed diagnosis of structural birth defects. Selection of the independent variables for the regression analysis was made based on a literature search and clinical experience. After checking for collinearity, the chosen variables underwent binary logistic regression analysis. Variable selection for inclusion in the multivariable model was based on statistical significance in bivariate analysis (*p*<0.25). A *p* value <0.05 was considered significant.

## Results

### Sociodemographic data

A total of 423 children with structural birth defects were enrolled. Males accounted for 358 (84.6%) of the study participants, with a male to female ratio of 5.5:1. Median age at the time of data collection was 2.12 years (IQR: 0.8–5.0 years, range: 2 days–5.5 years). Almost two-thirds of the children were urban residents. Nearly half of the parents or legal guardians were college graduates (table 1). Among children with structural birth defects, 42.0% were firstborns.

**Table 1:** Sociodemographic profile of children with structural birth defects and their parents or legal guardians

Variable	Frequency	Percentage
Sex		
Male	358	84.6
Female	65	15.4
Place of residence		
Urban	262	61.9
Rural	161	38.1
Parent/guardian literacy		
Illiterate	11	2.6
Can read and write only	6	1.4
Elementary school	75	17.7
Secondary school	126	29.8
College graduate	205	48.5
Monthly income		
<US$65	182	43.0
≥US$65	241	57.0

### Diagnosis

A total of 441 structural birth defects were diagnosed in 423 children, with 18 children having more than one anomaly. Of the identified birth defects (*n*=441), the three leading anomalies were hypospadias (133/441, 30.2%), undescended testes (95/441, 21.5%), and anorectal malformation (38/441, 8.6%) (table 2).

**Table 2:** Structural birth defects in study participants

Diagnosis	Frequency	Percentage
Hypospadias	133	30.2
Undescended testes	95	21.5
Anorectal malformation	38	8.6
Pelviureteric junction obstruction	33	7.5
Hirschsprung’s disease	29	6.6
Inguinal hernia	27	6.1
Cleft lip and cleft palate	16	3.6
Bladder exstrophy epispadias complex	8	1.8
Lymphatic malformation	7	1.6
Congenital pulmonary airway malformation	6	1.4
Posterior urethral valve	4	0.9
Esophageal atresia with tracheoesophageal fistula	4	0.9
Syndactyly	4	0.9
Vesicoureteral reflux	4	0.9
Congenital heart disease	3	0.7
Malrotation	3	0.7
Others	27	6.1
Total	441	100

### Timing of diagnosis

The majority of mothers (411, 97.2%) had antenatal follow-up and attended for an average of 5.3±1.7 visits. Antenatal ultrasound was performed in 384 (90.8%) of the mothers, with a mean number of ultrasound examinations of 3.3±1.4 times.

Among the 423 children, 77 (18.2%) had prenatally detectable birth defects. The top three anomalies were pelviureteric junction obstruction (33/77, 42.9%), bladder exstrophy (8/77, (10.4%), and lymphatic malformation (7/77, 9.1%). Among these, antenatal diagnosis was made in only 32 (41.6%); more than two-thirds of these children (24/32, 71.9%) had upper urinary tract dilatations. The remaining defects detected prenatally were lymphatic malformation (3/32, 9.4%), cystic thoracic masses (2/32, 6.2%), bladder exstrophy (2/32, 6.2%), and congenital heart disease (1/32, 3.1%).

Among the children with structural birth defects, 400 (94.6%) were delivered at a health facility, while the remaining 23 (5.4%) were born at home. Among 362 facility-born children with externally visible or clinically detectable defects, 104 (28.7%) were diagnosed before discharge. There was a history of neonatal intensive care unit admission in 67 (18.5%) children. Mean, median, and ranges for age at diagnosis for the five most common structural birth defects are described in table 3.

**Table 3:** Timing of diagnosis by age for the five most common structural birth defects

Diagnosis	Age at diagnosis			Children with delayed diagnosis (%)
	Mean±SD	Median age (IQR)	Range	
Hypospadias	1.1±2.2 years	14 days (1 hour–1 year)	1 hour–13.2 years	62.7
Undescended testes	3.4±3.5 years	2.6 years (0.5–5 years)	4 hours–14.1 years	88.4
Anorectal malformations	0.35±1.3 years	6.5 days (2–67.5 days)	1 hour–8.1 years	71.4
Pelviureteric junction obstruction	Antenatal: 29.3±3.9 weeks Postnatal: 6.5±4.7 years	Antenatal: 29 weeks (28–32) weeks Postnatal: 6 years (1.2–10) years	Antenatal: 20–36 weeks Postnatal: 2 days–13.7 years	51.5*
Hirschsprung’s disease	1.0±2.3 years	60 days (8 days–1 year)	1–12.2 years	86.2

* All children who were not diagnosed antenatally had delayed diagnosis.

Diagnosis of a structural birth defect was first made by a health professional in 243 (57.4%) cases, while parents or legal guardians and other family members were responsible for defect identification in 174 (41.1%) and 6 (1.4%) of the cases, respectively. Diagnosis of a structural birth defect was delayed in 274 (64.8%) children, while surgery was delayed in 235 (55.6%).

### Reasons for delayed diagnosis

Several reasons were given for the delayed diagnosis of structural birth defects by parents or legal guardians, including lack of awareness, delayed referral, false reassurance by a health professional, financial factors, inaccessibility of health facilities, false reassurance by a non-heath facilities, child wa asymptomatic and conflict zone residence. Seventy-four (17.5%) of parents provided more than one reason for delayed diagnosis or treatment.

### Factors associated with delayed diagnosis

Seven independent variables (male sex, income <US$65, rural residence, parental educational status, antenatal follow-up status, being firstborn, and being delivered at a health center) were selected for initial binary logistic analysis, based on literature and clinical experience/observation. Among these, four had a *p* value <0.25 and were further analyzed using multivariable logistic regression. This analysis found that being male (*p*=0.035, adjusted odds ratio (*aOR*)=1.81), having parents who are not college graduates (*p*=0.049, *aOR*=1.52), and being delivered at a health center (*p*<0.001, *aOR*=2.17) were associated with delayed diagnosis (table 4).

**Table 4:** Regression analysis of independent variables with a delayed diagnosis of structural birth defects

Variable	Diagnosis delayed		*P* value (binary)	*cOR*	*P* value (multivariable)	*aOR*	95% *CI*
	Yes	No					
Male sex	240	118	0.042	1.74	**0.035**	**1.81**	1.04 to 3.14
Income <US$65	116	66	0.633	0.91			
Rural residence	100	61	0.327	0.82			
Parents or legal guardians with no college degree	135	84	0.133	1.39	**0.049**	**1.52**	1.10 to 2.31
No antenatal follow-up	6	6	0.275	1.89			
Being firstborn	108	69	0.144	0.74	0.112	0.71	0.47 to 1.08
Being delivered at a health center	126	43	<0.001	2.07	**<0.001**	**2.17**	1.40 to 3.35

aOR, adjusted OR; cOR, crude OR.

A sensitivity analysis was performed by excluding the two dominant male genitourinary birth defects: hypospadias and undescended testes. On initial binary logistic regression, four variables (having parents or legal guardians who are not college graduates, no antenatal follow-up, being firstborn, and being delivered at a health center) exhibited a *p* value <0.25. These were subsequently analyzed using multivariable regression. The only variable that retained significance was being delivered at a health center (*p*<0.001, *aOR*=4.09, 95% *CI* 2.19 to 7.63). Having parents or legal guardians who are not college graduates (*p*=0.064) and being firstborn (*p*=0.054) showed a marginal association, with ORs that did not reach statistical significance (table 5).

**Table 5:** Regression analysis of independent variables with a delayed diagnosis of structural birth defects after excluding hypospadias and undescended testes

Variable	Delayed diagnosis		*P* value (binary)	*cOR*	*P* value (multivariable)	*aOR*	95% *CI*
	Yes	No					
Male sex	86	61	0.469	1.24			
Income <US$65	62	40	0.268	1.36			
Rural residence	49	43	0.35	0.77			
Having parents or legal guardians who are not college graduates	61	55	0.166	1.48	0.064	1.77	0.31 to 1.03
No antenatal follow-up	3	6	0.16	0.36	0.455	0.57	0.39 to 7.78
Being firstborn	44	43	0.12	0.65	0.054	0.55	0.13 to 2.51
Being delivered at a health center	65	21	<0.001	5.01	**<0.001**	**4.09**	2.19 to 7.63

aOR, adjusted OR; cOR, crude OR.

## Discussion

We analyzed the timing of diagnosis and factors associated with delayed presentation for 423 children with structural birth defects. The proportion of children who were diagnosed prenatally is critically low, given the high antenatal follow-up rates. Three-quarters of the children with physically detectable anomalies were also missed despite being born at health facilities. Lack of awareness about birth defects by parents or legal guardians, delayed referral and false reassurance from health professionals were the main reasons for delaying diagnosis.

Predominance of males in our study corresponds with other studies on children with congenital anomalies.^9–13^ This can be explained by the epidemiological observation that most of the structural birth defects in this study affect males disproportionately.

The type and pattern of structural birth defects vary among different studies. Genitourinary anomalies, like ours, took the major share in a study from Brazil, while central nervous system, cardiac, and musculoskeletal malformations were the leading defects in reports from Ghana, Canada, the UK and Cameroon.^10–12 14^ The average age at diagnosis of the three top common structural birth defects was compared with similar reports in sub-Saharan Africa; they fall within the same range, and there was a significant delay.^15 16^ This delay can be attributed to the complex interplay of socioeconomic and healthcare factors that impede timely diagnosis and access to specialized care for such children in sub-Saharan Africa. These barriers include economic hardship, cultural factors, lack of access to comprehensive antenatal and postnatal care, and shortage of trained healthcare professionals capable of identifying structural birth defects.

Prenatal detection of congenital malformations gives an opportunity to positively influence prenatal and postnatal management, survival, and morbidity, as well as to allow parental choice and psychological preparation. During the last decades, an increasing number of congenital malformations have been diagnosed prenatally using ultrasound investigation.^17^ A population-based study from Australia showed a fivefold increase in prenatal diagnosis of structural birth defects over 30 years, between 1980 and 2020.^18^

Antenatal detection of structural birth defects in our study was low, with rates half that of a report from Cameroon, three times lower than a study from Turkey, and eight times lower than a review from France and the wider European continent.^14 18 19^ The low figure in the face of an almost universal coverage of antenatal follow-up underlines the importance of integrating prenatal screening services using ultrasound into the antenatal care package. The impact of such intervention has been reported in one study from the Netherlands, which showed a 28% and 23% increase in the diagnosis of omphalocele and gastroschisis, respectively; this was after the introduction of a national screening program for birth defects using prenatal ultrasound.^20^

In several LMICs, the diagnosis of structural birth defects, even after birth, is often delayed. One scoping review, which studied five structural birth defects in children from 12 African countries, revealed a pronounced delay in the timing of diagnosis and surgical intervention.^15^ Our study also demonstrated delayed diagnosis in two-thirds of children. Furthermore, despite being delivered in a health facility, the diagnosis of three-quarters of children born with overt or physically detectable defects was missed. This significant missed opportunity underscores the critical need for a neonatal screening program that involves a comprehensive clinical examination of all newborns before they leave the health facility. This strategy is likely to be effective given the high (94.6%) health facility delivery rate in this study.

Delayed diagnosis of structural birth defects is a significant concern as it hampers timely intervention and treatment, which are crucial for improving the health outcomes of affected children. The association between late diagnosis and surgical intervention for structural birth defects with increased mortality has been demonstrated by several reports from sub-Saharan Africa.^21–23^

The most frequently mentioned reasons for late diagnosis of structural birth defects in the Global South are limited access to healthcare facilities, insufficient medical resources, and a lack of trained healthcare professionals equipped to identify and manage these conditions promptly. In this study, the predominance of delayed referral and incorrect reassurance by health professionals as key contributors to delayed diagnosis of structural birth defects, surpassing barriers such as facility access and financial constraints, was an unexpected finding. This evidence calls for robust capacity-building sessions for healthcare workers, particularly on the identification and optimal time for management of structural birth defects.

This study has identified significant missed opportunities for the diagnosis of structural birth defects during both prenatal and postnatal periods. Strategies suggested to overcome these gaps include introducing screening programs during pregnancy and before discharging newborns from health facilities and providing comprehensive capacity-building training for health professionals about structural birth defects. In this study, the diagnosis of a structural birth defect in children who were born at health centers was twice as high. This difference may be attributed to variations in staff profiles between health centers and hospitals. In health centers, births are almost exclusively attended by nurses and midwives, whereas in hospitals, physicians—ranging from general practitioners to consultant obstetricians—are primarily responsible. Physicians are generally expected to have greater expertise in recognizing structural birth defects. In addition, a greater share of mothers attended their antenatal follow-ups and delivered in health centers. Attention should be given to empower these primary healthcare units to implement the recommended strategies.

### Conclusion and recommendations

There is a significant delay in diagnosing structural birth defects during both the antenatal and postnatal periods. Findings of this study imply that these missed opportunities can be addressed through comprehensive screening programs, capacity-building sessions for healthcare workers, and raising public awareness on structural birth defects. Crucially, implementation of these strategies should prioritize empowering primary healthcare units, particularly health centers, to facilitate the early diagnosis of structural birth defects. While this study provides important insight and transferable lessons for other low-resource urban settings where primary care and referral systems function similarly, large-scale population studies are recommended to accurately define the extent and reasons for delayed diagnosis of structural birth defects.

### Limitations of this study

This study has several limitations. First, as this is a facility-based cross-sectional study conducted in a few public referral hospitals in Addis Ababa, the findings reflect a highly selected case mix and may not be generalizable to children with structural birth defects in community or rural settings. We recognize that the predominance of male genitourinary anomalies, particularly hypospadias and undescended testes, has influenced age at diagnosis and prevalence of delay. This also reflects the case mix in participating hospitals, where roughly 60% of pediatric elective surgeries are for genitourinary conditions. This has resulted in the relative scarcity of anomalies, which will result in neonatal mortality if not detected immediately after birth. In addition, the facility-based context itself, including referral pathways and institutional design, may have influenced both the timing of diagnosis and the reasons for delay. Moreover, information on reasons for delayed diagnosis relied entirely on parental or guardian reports, which may be subject to recall bias and social desirability bias.

## Supplementary material

10.1136/wjps-2026-001186 online supplemental file 1

10.1136/wjps-2026-001186 online supplemental file 2

## Data Availability

Data are available upon reasonable request.
