# Supplementary material for: Timing of diagnosis and factors associated with delayed detection of structural birth defects in children: a multicenter cross-sectional study from Addis Ababa, Ethiopia
Source: World J Pediatr Surg. 2026 Aug 12;9(4):e001186. doi: 10.1136/wjps-2026-001186 (PMC13475084; doi:10.1136/wjps-2026-001186)
Supplement: Supplementary file 1 [file wjps-9-4-s001.pdf]

Supplemental File 1

System-Based Description of Structural Birth Defects

Category	Description	List of structural birth defects
Prenatally detectable	These structural birth defects are considered amenable to detection during pregnancy using ultrasound	Head and neck<br>- Neural tube defects, cleft lip and palate, neck masses<br>Chest<br>- Congenital diaphragmatic hernia, esophageal atresia, cystic pulmonary malformations, duplication cysts, major congenital heart defects (eg, hypoplastic left heart, tetralogy of Fallot)<br>Abdomen<br>- Omphalocele, gastroschisis, duodenal atresia, jejuno-ileal atresia<br>Genitourinary system<br>- Upper urinary tract anomalies manifested by hydronephrosis (multicystic dysplastic kidney), Pelviureteric junction obstruction, vesicoureteral junction obstruction or reflux, ureteric duplication and ectopia, ureterocele, posterior urethral valve, bladder exstrophy<br>Skeletal<br>- Limb reduction defects, absence of limbs, club foot, polydactyly<br>Nervous system<br>- Neural tube defects
Overt or physically detectable	These structural birth defects are visible or can be identified through physical examination right after birth	Head and neck<br>- encephalocele, cleft lip and palate, neck masses<br>Chest<br>- chest wall deformity (pectus carinatum, pectus excavatum), most of the congenital heart diseases<br>Abdomen<br>- anorectal malformations, omphalocele, gastroschisis, inguinal hernia, prune belly syndrome<br>Genitourinary system<br>- Hypospadias, undescended testes, bladder exstrophy, DSD<br>Skeletal<br>- Limb reduction defects, absence of limbs, club foot, polydactyly<br>Nervous system<br>- Myelomeningocele

Supplemental File 1

System-Based Description of Structural Birth Defects

Structural birth defects that cause early symptoms	These structural birth defects cause physical symptoms within the first few days after birth, mostly in the first week of life	Head and neck<br>- Bilateral choanal atresia<br>Chest<br>- esophageal atresia, congenital diaphragmatic hernia (two-thirds of newborns), most cystic pulmonary malformations and duplication cysts, major congenital heart defects (eg, hypoplastic left heart, tetralogy of Fallot)<br>Abdomen<br>- duodenal atresia, jejuno-ileal atresia, anorectal malformations, Hirschsprung's disease, malrotation with midgut volvulus, omphalocele, gastroschisis<br>Genitourinary system<br>- posterior urethral valve, bilateral renal agenesis or dysplasia<br>Nervous system<br>- Ruptured myelomeningocele
No external signs or not symptomatic	These structural birth defects have no external stigmata that can be used for identification. They usually don't manifest during neonatal and infancy periods and may be incidentally found or may cause symptoms later during childhood	Head and neck<br>- unilateral choanal atresia<br>Chest<br>- congenital diaphragmatic hernia (one-third of newborns), minor congenital heart defects (eg, Small VSD, ASD)<br>Genitourinary system<br>- mild to moderate upper urinary tract obstructions<br>Nervous system<br>- Tethered cord, occult spinal dysraphism
