# Supplementary material for: Timing of diagnosis and factors associated with delayed detection of structural birth defects in children: a multicenter cross-sectional study from Addis Ababa, Ethiopia
Source: World J Pediatr Surg. 2026 Aug 12;9(4):e001186. doi: 10.1136/wjps-2026-001186 (PMC13475084; doi:10.1136/wjps-2026-001186)
Supplement: Supplementary file 2 [file wjps-9-4-s002.pdf]

Supplemental File 2

Case-by-case definition of delayed diagnosis of structural birth defects

Diagnosis	Definition of delayed diagnosis
Hypospadias	- Neonates born at a health facility- diagnosis not made before the discharge of the newborn<br>- Neonates born at home- diagnosis beyond 1 week of age
Undescended testes	- Neonates born at a health facility- diagnosis not made before the discharge of the newborn<br>- Neonates born at home- diagnosis beyond 1 week of age
Anorectal malformation	- Neonate born at a health facility- diagnosis not made before the discharge of the newborn<br>- Neonate born at home- diagnosis beyond 1 week of age
Pelviureteric junction obstruction	- Neonates whose mother had antenatal follow-up-diagnosis not made before birth<br>- Neonates whose mother had no antenatal follow-up and/or those born at home- diagnosis beyond 28 days of life
Hirschsprung's disease	- Neonate born at a health facility- diagnosis beyond 1 week of age<br>- Neonate born at home- diagnosis beyond 1 week of age
Inguinal hernia	- Neonate born at a health facility- diagnosis not made before the discharge of the newborn<br>- Neonate born at home- diagnosis beyond 1 week of age
Cleft lip and cleft palate	- Neonate born at a health facility- diagnosis not made before the discharge of the newborn<br>- Neonate born at home- diagnosis beyond 1 week of age

Bladder exstrophy epispadias complex	- Neonates whose mother had antenatal follow-up-diagnosis not made before birth<br>- Neonates whose mother had no antenatal follow-up and/or those born at home- diagnosis beyond 1 week of age
Lymphatic malformation	- Neonate born at a health facility- diagnosis not made before the discharge of the newborn<br>- Neonate born at home- diagnosis beyond 1 week of age
Congenital pulmonary airway malformation	- Neonates whose mother had antenatal follow-up-diagnosis not made before birth<br>- Neonates whose mother had no antenatal follow-up and/or those born at home- diagnosis beyond 28 days of life
Posterior urethral valve	- Neonates whose mother had antenatal follow-up-diagnosis not made before birth<br>- Neonates whose mother had no antenatal follow-up and/or those born at home- diagnosis beyond 28 days of life
Esophageal atresia with or without tracheoesophageal fistula	- Neonates whose mother had antenatal follow-up-diagnosis not made before birth<br>- Neonates whose mother had no antenatal follow-up and/or those born at home- diagnosis beyond 1 week of age
Syndactyly	- Neonate born at a health facility- diagnosis not made before the discharge of the newborn<br>- Neonate born at home- diagnosis beyond 1 week of age

Vesicoureteral reflux	- Neonates whose mother had antenatal follow-up-diagnosis not made before birth<br>- Neonates whose mother had no antenatal follow-up and/or those born at home- diagnosis beyond 28 days of life
Congenital heart disease	- Neonates whose mother had antenatal follow-up (for major cyanotic heart defects) -diagnosis not made before birth<br>- Neonate born at a health facility with minor cardiac defects- diagnosis beyond 28 days<br>- Neonates whose mother had no antenatal follow-up and/or those born at home- diagnosis beyond 28 days
Malrotation	- Neonate born at a health facility- diagnosis beyond 1 week of age<br>- Neonate born at home- diagnosis beyond 1 week of age
